# Unveiling the Antifouling Performance of Different Marine Surfaces and Their Effect on the Development and Structure of Cyanobacterial Biofilms

**DOI:** 10.3390/microorganisms9051102

**Published:** 2021-05-20

**Authors:** Sara I. Faria, Rita Teixeira-Santos, Maria J. Romeu, João Morais, Ed de Jong, Jelmer Sjollema, Vítor Vasconcelos, Filipe J. Mergulhão

**Affiliations:** 1 LEPABE—Department of Chemical Engineering, Faculty of Engineering, University of Porto, Rua Dr. Roberto Frias, 4200-465 Porto, Portugal; sisf@fe.up.pt (S.I.F.); ritadtsantos@fe.up.pt (R.T.-S.); mariaromeu@fe.up.pt (M.J.R.); 2 CIIMAR—Interdisciplinary Centre of Marine and Environmental Research, University of Porto, Terminal de Cruzeiros do Porto de Leixões, Avenida General Norton de Matos, S/N, 4450-208 Matosinhos, Portugal; jmorais@ciimar.up.pt (J.M.); vmvascon@fc.up.pt (V.V.); 3 Department of Biomedical Engineering, University of Groningen, University Medical Centre Groningen, A. Deusinglaan 1, 9713 AV Groningen, The Netherlands; e.d.de.jong@umcg.nl (E.d.J.); j.sjollema@umcg.nl (J.S.); 4 FCUP—Faculty of Sciences, University of Porto, Rua do Campo Alegre, 4069-007 Porto, Portugal

**Keywords:** biofilm development, biofilm structure, coccoid cyanobacteria, marine surface materials, epoxy resin, silicone hydrogel

## Abstract

Since biofilm formation by microfoulers significantly contributes to the fouling process, it is important to evaluate the performance of marine surfaces to prevent biofilm formation, as well as understand their interactions with microfoulers and how these affect biofilm development and structure. In this study, the long-term performance of five surface materials—glass, perspex, polystyrene, epoxy-coated glass, and a silicone hydrogel coating—in inhibiting biofilm formation by cyanobacteria was evaluated. For this purpose, cyanobacterial biofilms were developed under controlled hydrodynamic conditions typically found in marine environments, and the biofilm cell number, wet weight, chlorophyll *a* content, and biofilm thickness and structure were assessed after 49 days. In order to obtain more insight into the effect of surface properties on biofilm formation, they were characterized concerning their hydrophobicity and roughness. Results demonstrated that silicone hydrogel surfaces were effective in inhibiting cyanobacterial biofilm formation. In fact, biofilms formed on these surfaces showed a lower number of biofilm cells, chlorophyll *a* content, biofilm thickness, and percentage and size of biofilm empty spaces compared to remaining surfaces. Additionally, our results demonstrated that the surface properties, together with the features of the fouling microorganisms, have a considerable impact on marine biofouling potential.

## 1. Introduction

Marine biofouling is the attachment of undesirable molecules and micro- and macroorganisms to submerged surfaces, posing serious economic and environmental implications. Biofouling on ship hulls causes an increase in frictional drag, which leads to higher fuel consumption, maintenance costs, and downtimes [1,2,3]. Moreover, submerged marine facilities and equipment can be damaged by biofouling, representing additional costs for marine industries [1]. Furthermore, this natural process allows the bio-invasion of exotic species whenever fouling organisms travel in vessel hulls (ships, yachts, or sailing boats) across different geographic areas, compromising the conservation of marine ecosystems [4,5]. For these reasons, there is a clear need to develop more efficient antifouling coatings and understand their interactions with microfouling organisms during biofilm formation, since this is the initial colonization stage.

Biofouling occurs spontaneously via the adhesion of microfouling organisms (e.g., cyanobacteria and diatoms) to underwater surfaces with consequent biofilm formation, which builds the basis for the later settlement of macrofouling organisms (e.g., bryozoans, mollusks, polychaeta, tunicates, coelenterates, or fungi) [6]. This is a dynamic process that involves several consecutive steps and is modulated by different factors, such as the surface properties, hydrodynamic conditions, and microbial composition [7]. At the initial stages of biofouling, the physicochemical properties of marine surfaces, including surface free energy, roughness, and hydrophobicity may have a significant impact on the rate and extent of microorganisms adhesion and biofilm formation [8,9]. Moreover, microorganisms live in microenvironments subject to external factors that condition local nutrient transport and chemical gradients, creating specialized niches and shaping the biofilm structure [10]. The spatial organization of microorganisms can influence emergent phenomena like quorum sensing, intracellular communication, and biofilm formation, conferring them greater resistance to mechanical and chemical stresses (e.g., fluid shear, detergents, and antifouling compounds) [11].

Although it is known that microbial biofilms are complex systems that shape, and are shaped by, their local microenvironments [10], there are few studies about how marine surfaces influence microfouler attachment and biofilm formation [12].

In this study, the long-term performance of five surface materials—glass, perspex, polystyrene, epoxy-coated glass, and a silicone hydrogel coating—in inhibiting or delaying biofilm formation by microfoulers was evaluated. Glass, perspex, and polystyrene materials are commonly found on different marine facilities and equipment, including underwater windows of boats, aquaculture systems, flotation spheres, moored buoys, underwater cameras, measuring devices or sensors, pontoons, and floating docks [13,14]. In turn, polymer epoxy resin and silicone hydrogel are two commercial marine coatings; the first is used to coat the hulls of small recreation vessels (e.g., powerboats, yachts, and sailing boats) [15,16], while the second is frequently used to coat ship hulls, marine water inlet piping, and grids in power stations [17].

Although the presented materials are typically found in marine environments, the microfouler response to these surfaces is not adequately characterized, and their effect on the development and structure of marine biofilms is unexplored. Hence, the present study aimed to evaluate the long-time performance of these surface materials against biofilm formation by one of the most common microfouling organisms [18,19], cyanobacteria, under defined hydrodynamic conditions, to estimate their antifouling performance and to assess their effects on biofilm architecture in conditions mimicking marine settings.

## 2. Materials and Methods

### 2.1. Surface Preparation

Cyanobacterial biofilm formation was studied using five different marine surfaces, glass, perspex, polystyrene, epoxy-coated glass, and a silicone hydrogel coating.

Glass, perspex, and polystyrene surfaces were cut into squares (1 × 1 cm) designated by coupons. The epoxy resin and silicone hydrogel coatings were prepared using glass coupons as a substrate, as described below.

Glass (Vidraria Lousada Lda, Lousada, Portugal), perspex (Neves & Neves Lda, Porto, Portugal), and polystyrene (VWR, International, Carnaxide, Portugal) coupons were cleaned and disinfected by immersion in a 2% (*v*/*v*) TEGO 2000^®^ solution (an amphoteric disinfectant; JohnsonDiversey, Northampton, United Kingdom), for 20 min under agitation (150 rpm) [20,21]. Subsequently, coupons were washed with sterile distilled water to remove possible remains of the disinfectant solution, air-dried, and sterilized by autoclaving at 121° for 15 min (glass) or UV radiation for 30 min (perspex and polystyrene).

Epoxy resin- and silicone hydrogel-coated surfaces were prepared using glass as a substrate following the protocol described by Faria et al. [17]. Briefly, 70 µL of epoxy resin (HB Química, Porto, Portugal) was deposited on the top of glass coupons by spin coating (Spin150 PolosTM, Paralab, Portugal) at 6000 rpm, with increments of 1000 rpm, for 40 s. Afterward, surfaces were dried in two sequential steps (12 h at room temperature and 3 h at 60 °C) and sterilized by immersion in 70% (*v*/*v*) ethanol (VWR International S.A.S., Fontenay-sous-Bois, France) for 20 min [7]. The silicone hydrogel surfaces (HEMPASIL X3+ 87500, Copenhagen, Denmark) were prepared using conventional brush painting following the recommendations of the manufacturer and sterilized by UV radiation for 30 min [17].

Before the biofilm formation experiments, the initial weight of each coupon was determined.

### 2.2. Surface Characterization

#### 2.2.1. Atomic Force Microscopy (AFM)

AFM studies were performed using a Bruker Catalyst microscope in contact mode with a DNP-D cantilever with a spring constant of 0.06 N/m (Bruker Billerica, MA, USA). The surface roughness was determined from three random areas (75 × 75 µm) on three samples at room temperature. The scan speed was set to 1 Hz. Surface roughness calculations and 2D images were made using the Nanoscope Analysis Software from Bruker. The roughness height parameter determined was the average roughness (*R_a_*).

#### 2.2.2. Thermodynamic Analysis

The hydrophobicity of the surfaces and cyanobacteria cells was determined by contact angle measurement [22] and, subsequently, estimated using the van Oss approach [23].

Cyanobacterial substrata were prepared by filtering cell suspensions containing 1 × 10^9^ cells·mL^−1^ using cellulose membranes following the protocol developed by Busscher et al. [24]. The contact angles of materials and cyanobacteria cells were determined automatically at 25 ± 2 °C by the sessile drop method in a contact angle meter (Dataphysics OCA 15 Plus, Filderstadt, Germany) using water, formamide, and α-bromonaphthalene as reference liquids, in three independent assays. For each experiment, at least 25 measurements were performed.

Water contact angles (*θ*_w_) indicate the surface hydrophobicity (*θ*_w_ < 90° indicates that a surface is hydrophilic, while *θ*_w_ > 90° indicates that it is hydrophobic) [22].

In turn, based on the van Oss approach [23], the total surface free energy (*γ^TOT^*) of a pure substance results from the sum of the apolar Lifshitz–van der Waals component of the surface free energy (*γ^LW^*) and the polar Lewis acid–base component (*γ^AB^*).

(1) $$\gamma^{TOT}=\gamma^{LW}+\gamma^{AB}.$$

The polar AB component comprises the electron acceptor, *γ*^+^, and electron donor, *γ*^−^, parameters and is given by

(2) $$\gamma^{AB}=2\sqrt{\gamma^{+}\gamma^{-}}.$$

The surface free energy components of a solid surface (*s*) are obtained by measuring the contact angles (*θ*) with three different liquids (*l*) with known surface tension components [25], followed by the simultaneous resolution of three equations of the following type:

(3) $$(1+\cos\theta )\gamma_{l}=2\left(\sqrt{\gamma_{s}^{LW}\gamma_{l}^{LW}}+\sqrt{\gamma_{s}^{+}\gamma_{l}^{-}}+\sqrt{\gamma_{s}^{+}\gamma_{l}^{-}}\right).$$

The degree of hydrophobicity of a given surface is expressed as the free energy of interaction (Δ*G*, mJ·m^−2^) between two entities of that surface immersed in polar liquid (such as water (*w*) as a model solvent). Therefore, *ΔG* is calculated using the following equation:

(4) $$\Delta G=-2\;{\left(\sqrt{\gamma_{s}^{LW}}-\sqrt{\gamma_{w}^{LW}}\right)}^{2}-4\;\left(\sqrt{\gamma_{s}^{+}\gamma_{w}^{-}}+\sqrt{\gamma_{s}^{-}\gamma_{w}^{+}}-\sqrt{\gamma_{s}^{+}\gamma_{s}^{-}}-\sqrt{\gamma_{w}^{+}\gamma_{w}^{-}}\right).$$

According to this approach, if the interaction between the two entities is stronger than the interaction of each one with water (Δ*G* < 0 mJ·m^−2^), the material is considered hydrophobic (free energy of interaction is attractive); contrarily, if Δ*G* > 0 mJ·m^−2^, the material is hydrophilic (free energy of interaction is repulsive) [26].

When studying the interaction (free energy of adhesion) between the surface (*s*) and cyanobacteria cells (*b*), the total interaction energy, Δ*G^Adh^*, can be expressed as

(5) $$\Delta G^{Adh}=\gamma_{sb}^{Lw}-\gamma_{sw}^{Lw}-\gamma_{bw}^{Lw}+2\left[\sqrt{\gamma_{w}^{+}}\left(\sqrt{\gamma_{s}^{-}}+\sqrt{\gamma_{b}^{-}}-\sqrt{\gamma_{w}^{-}}\right)+\sqrt{\gamma_{w}^{-}}\left(\sqrt{\gamma_{s}^{+}}+\sqrt{\gamma_{b}^{+}-\sqrt{\gamma_{w}^{+}}}\right)-\sqrt{\gamma_{s}^{+}\gamma_{b}^{-}}-\sqrt{\gamma_{s}^{-}\gamma_{b}^{+}}\right].$$

Thermodynamically, if Δ*G^Adh^* < 0 mJ·m^−2^, the adhesion of cyanobacteria to the material is favored; on the other hand, the adhesion is thermodynamically not favorable when Δ*G^Adh^* > 0 mJ·m^−2^.

### 2.3. Marine Organisms and Growth Conditions

Three coccoid cyanobacteria isolates, *Synechocystis salina* LEGE 00041, *Cyanobium* sp. LEGE 06098, and *Cyanobium* sp. LEGE 10375, from the Blue Biotechnology and Ecotoxicology Culture Collection (LEGE-CC), deposited at the Interdisciplinary Centre of Marine and Environmental Research (CIIMAR), Matosinhos, Portugal, were used in this study.

*S. salina* LEGE 00041 was isolated from a tide pool, on the intertidal zone, in June 2000, at Espinho beach (41.00847 N 8.646958 W) located on the north coast of Portugal. *Cyanobium* sp. LEGE 06098 was originally obtained from an intertidal zone, in a green macroalga, collected in July 2006, at Martinhal beach (37.01869 N 8.926714 W) located in Vila do Bispo, Portugal. *Cyanobium* sp. LEGE 10375 was isolated from the intertidal zone, in a marine sponge, collected in October 2010, at São Bartolomeu do Mar beach (41.57378 N 8.798556 W) located in Esposende, Portugal [27]. The organisms used in this study comprise cyanobacterial isolates from different geographical locations and taxonomic genera with the aim of assessing the influence of different genotypic and phenotypic profiles on surface material performance.

Cyanobacterial isolates were grown in Z8 medium [28] supplemented with 25 g·L^−1^ of synthetic sea salts (Tropic Marin) and B12 vitamin (Sigma Aldrich, Merck, Saint Louis, MO, USA), under 14 h light (10–30 mol photons·m^−2^·s ^−1^, λ = 380–700 nm)/10 h dark cycles at 25 °C [7].

### 2.4. Biofilm Formation Assays

Cyanobacterial biofilm formation assays were performed using 12-well plates (VWR International, Carnaxide, Portugal) under controlled hydrodynamic conditions. Sterilized coupons of glass, perspex, polystyrene, epoxy-coated glass, and silicone hydrogel coating were fixed to the microplate wells using transparent double-sided adhesive tape. Then, 3 mL of cyanobacterial suspension at a final concentration of 1 × 10^8^ cells·mL^−1^ was added to each well, and plates were incubated at 25 °C in an orbital shaker with a 25 mm diameter (Agitorb 200ICP, Norconcessus, Ermesinde, Portugal) at 185 rpm, under alternate light cycles of 14 h light (10–30 mol photons·m^−2^·s^−1^)/10 h dark. According to previous computational fluid dynamic studies using this type of incubator [14], a shaking frequency of 185 rpm corresponds to an average shear rate of 40 s^−1^ and a maximum of 120 s^−1^, which encompasses the shear rate estimated for a ship in a harbor (50 s^−1^) [29].

Biofilm formation experiments were monitored for 7 weeks (49 days) since this period corresponds on average to half of the minimal economically viable interval accepted for the maintenance of underwater systems [15] and hull cleaning [30,31]. During this period, the culture medium was replaced twice a week. On day 49, two coupons of each material were removed and gently rinsed in a sterile sodium chloride solution (8.5 mg·mL^−1^) to remove loosely attached cyanobacteria. Subsequently, coupons were analyzed concerning the number of biofilm cells, biofilm wet weight, chlorophyll *a* content, and biofilm thickness and structure.

Biofilm experiments were performed in duplicate and in three independent assays.

#### 2.4.1. Biofilm Cell Counting

To determine the number of biofilm cells, coupons were dipped in 2 mL of 8.5 mg·mL^−1^ sodium chloride solution (VWR International, Carnaxide, Portugal) and vortexed for 3 min at maximum power to release cells. Subsequently, 10 μL of each cellular suspension was placed on each side of the Neubauer counting chamber and observed in a microscope (Nikon Eclipse LV100 microscope, Nikon Corporation, Tokyo, Japan). In order to confirm complete cyanobacterial detachment, coupons were also analyzed under the microscope.

#### 2.4.2. Biofilm Wet Weight

To evaluate the biofilm wet weight, coupons were removed from the microplate wells using a sterile tweezer and weighted. The biofilm wet weight was determined by the difference between the initial weight of coupons (before inoculation) and the weight measured on day 49.

#### 2.4.3. Chlorophyll *a* Content

Cyanobacterial cells (detached from coupons as described in Section 2.4.1) were harvested by centrifugation at 3202× *g*, for 5 min at room temperature, and the supernatant was discarded. As chlorophyll pigments are light-sensitive, their extraction was performed in the dark according to the procedure described by Romeu et al. [14]. Briefly, 2 mL of 99.8% methanol (VWR International, Carnaxide, Portugal) was added to the pellet and incubated at 4 °C, for 24 h. The absorbances at 750 nm (turbidity), 665 nm (chlorophyll *a*), and 652 nm (chlorophyll *b*) were measured on a V-1200 spectrophotometer (VWR International China Co., Ltd., Shanghai, China). The chlorophyll *a* concentration (μg·cm^−2^) was calculated using Equation (6).

Chl *a* (μg·mL^−1^) = 16.29 × A^665^ − 8.54 × A^652^. (6)

#### 2.4.4. Biofilm Thickness and Structure

The evaluation of biofilm thickness and structure was performed on day 49 through optical coherence tomography (OCT) using a Thorlabs Ganymede Spectral Domain Optical Coherence Tomography system with a central wavelength of 930 nm (Thorlabs GmbH, Dachau, Germany). Before biofilm analysis, the culture medium was carefully removed from the microplate wells, coupons were washed once, and wells filled with 3 mL of 8.5 g·L^−1^ sodium chloride sterile solution. Images from cyanobacterial biofilms developed on studied surfaces were captured and analyzed as previously described by Romeu et al. [14]. For each coupon, 2D and 3D imaging were performed with a minimum of three fields of view, to ensure the accuracy and reliability of the obtained results. For image analysis, the bottom of the biofilm was determined as the best-fitting parabole and hyperboloid, in 2D and 3D images, respectively, that connected the white pixels resulting from light reflection on the substratum surface. A gray-value threshold that separates the biofilm from the background was calculated on the basis of the gray-value histogram of the entire image [32]. The upper contour line of the biofilm was defined as those pixels in the image that have a gray value just higher than the gray-value threshold and are connected to the biofilm bottom. Objects not connected to the bottom were rejected from the biofilm structure, and the mean biofilm thickness was calculated as a function of the number of pixels between the bottom of the biofilm and the upper contour line for each vertical line in the image.

### 2.5. Statistical Analysis

Descriptive statistics were used to calculate the mean and standard deviation for the contact angles, surface roughness, number of biofilm cells, biofilm wet weight, chlorophyll *a* content, biofilm thickness, and percentage and size of biofilm empty spaces.

Differences in the number of biofilm cells, biofilm wet weight, chlorophyll *a* content, biofilm thickness, and biofilm empty spaces obtained for tested surfaces (glass, perspex, polystyrene, epoxy-coated glass, and silicone hydrogel coating) were evaluated using Kruskal–Wallis and Mann–Whitney tests since the variables were not normally distributed.

Statistically significant differences were considered for *p*-values <0.05. Letters were assigned in alphabetic order from the highest to the lowest value (from *a* to *e*) for each surface. These assignments were made as long as statistically significant differences existed between the biofilms.

Data analysis was performed using the IBM SPSS Statistics version 24.0 for Windows (IBM SPSS, Inc., Chicago, IL, USA).

## 3. Results

In this study, the antifouling performance of five different marine surface materials, glass, perspex, polystyrene, epoxy-coated glass, and a silicone hydrogel coating, was evaluated through the analysis of cyanobacterial biofilms formation on those substrates for 49 days under controlled hydrodynamic conditions.

### 3.1. Surface Characterization of Materials and Cyanobacterial Isolates

Since it is known that surface properties influence cell adhesion and subsequent biofilm formation [33,34], surface materials were first analyzed regarding their hydrophobicity, topography, and roughness. Table 1 presents the contact angles, hydrophobicity, and roughness values for the tested materials. The hydrophobicity was evaluated by contact angle measurement and based on the van Oss approach [23]. Considering that water contact angles (*θ*_w_) values <90° and free energy of interaction (Δ*G*) values >0 mJ·m^−2^ indicate that a surface is hydrophilic, both measures showed that glass is the most hydrophilic material (*θ_w_* = 27.8° ± 4.0°; ∆*G* = 32.5 mJ·m^−2^) followed by epoxy-coated glass (*θ_w_* = 69.4° ± 3.0°; ∆*G* = −6.7 mJ·m^−2^), perspex (*θ_w_* = 72.6° ± 3.2°; ∆*G* = −42.7 mJ·m^−2^), and polystyrene (*θ_w_* = 77.9° ± 3.6°; ∆*G* = −43.8 mJ·m^−2^). Moreover, the hydrophobic behavior of the silicone hydrogel coating was also demonstrated by the water contact angle (*θ_w_* = 108.4° ± 3.5°; *θ*_w >_ 90°), as well as the degree of hydrophobicity (Δ*G* = −55.8 mJ·m^−2^; *∆G* < 0 mJ·m^−2^).

**Figure 1:**
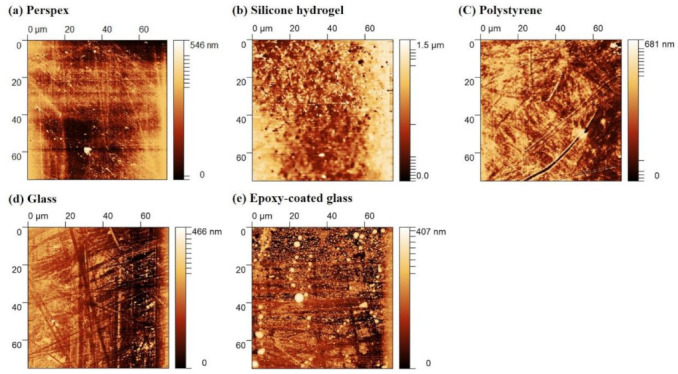
Two-dimensional AFM images of perspex (**a**), silicone hydrogel coating (**b**), polystyrene (**c**), glass (**d**), and epoxy-coated glass (**e**) surfaces with a scan range of 75 × 75 μm (contact mode). The color bar corresponds to the *z*-range (surface height range) of the respective image.

The surface topography and roughness of the five materials were evaluated by AFM in contact mode as these parameters are directly related to cell adhesion [35,36]. The topography images revealed that perspex and glass are the most homogeneous and smooth materials (Figure 1a,d). In fact, these surfaces displayed, on average, a lower roughness value (*R_a_* = 6.2 nm) compared to the other surfaces. The polystyrene and epoxy-coated glass showed *R_a_* values of 10.1 and 13.4 nm, respectively (Table 1). In opposition, the silicone hydrogel coating registered the highest *R_a_* value (49.7 nm).

Because cell adhesion and biofilm formation are also influenced by the physicochemical properties of the microorganisms [37,38], the water contact angles and degree of hydrophobicity of cells were also assessed (Table 2).

*Cyanobium* sp. LEGE 10375 showed the highest Δ*G* value and, thus, this strain is relatively more hydrophilic (∆*G* > 0 mJ·m^−2^) than the other cyanobacteria (Δ*G*_10375_ = 63.2 mJ·m^−2^ > Δ*G*_06098_ = 53.1 mJ·m^−2^ > Δ*G*_00041_ = 42.6 mJ·m^−2^).

As the free energy of the interaction between cyanobacterial isolates and tested surfaces can estimate the extent of cell adhesion [37], it was calculated and the results are presented in Table 3. The values of free energy of adhesion (Δ*G^Adh^*) obtained for the different cyanobacteria strains were similar for the same material and indicated that cell adhesion on the silicone hydrogel coating and perspex (lower Δ*G^Adh^* values) is thermodynamically more favorable compared to other materials, particularly to glass.

### 3.2. Quantification of Biofilms Developed on Tested Surfaces

**Figure 2:**
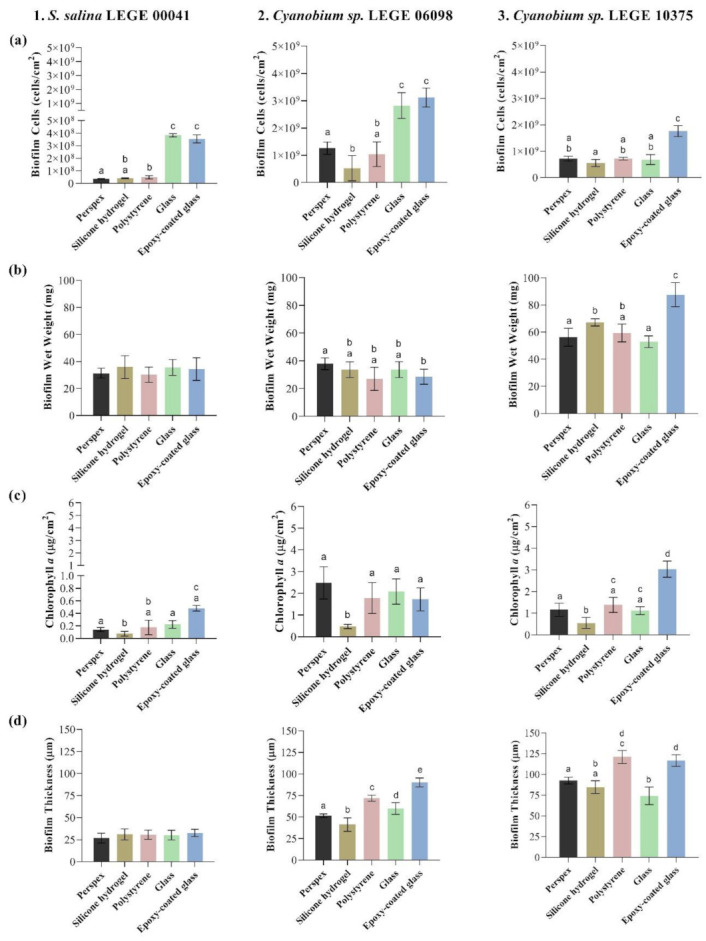
Biofilm development of *S. salina* LEGE 00041 (**1**), *Cyanobium* sp. LEGE 06098 (**2**), and *Cyanobium* sp. LEGE 10375 (**3**) on perspex ■, silicone hydrogel coating ■, polystyrene ■, glass ■, and epoxy-coated glass ■ surfaces after 49 days. The analyzed parameters refer to the number of biofilm cells (**a**), biofilm wet weight (**b**), chlorophyll *a* content (**c**), and biofilm thickness (**d**). Error bars indicate the standard error of the mean. For each cyanobacterial isolate, different lowercase letters indicate significant differences between surfaces with a confidence level greater than 95% (*p* < 0.05).

Cyanobacterial biofilm formation on the tested surfaces was assessed on day 49 through an analysis of the number of biofilm cells, biofilm wet weight, chlorophyll *a* content, and biofilm thickness (Figure 2) in order to evaluate the performance of tested surface materials. Regardless of surface material, *S. salina* LEGE 00041 had a lower biofilm-forming capacity than the other cyanobacteria, as demonstrated by the low number of adhered cells.

Concerning the number of biofilm cells (Figure 2a), the glass and epoxy-coated glass surfaces showed, on average, a higher number of attached cells for *S. salina* LEGE 00041 (3.83 × 10^8^ ± 1.27 × 10^7^ and 3.55 × 10^8^ ± 3.24 × 10^7^ cells·cm^−2^, respectively; Figure 2(1a)) and *Cyanobium* sp. LEGE 06098 (2.82 × 10^9^ ± 4.72 × 10^8^ and 3.12 × 10^9^ ± 3.49 × 10^8^ cells·cm^−2^, respectively; Figure 2(2a)). *Cyanobium* sp. LEGE 10375 displayed, on average, a higher number of biofilm cells on epoxy-coated glass surfaces (1.76 × 10^9^ ± 2.08 × 10^8^ cells·cm^−2^; Figure 2(3a)). Conversely, the silicone hydrogel-coated surfaces registered, on average, a lower number of biofilm cells for *Cyanobium* sp. LEGE 06098 (5.24 × 10^8^ ± 4.65 × 10^8^ cells·cm^−2^; Figure 2(2a)) and *Cyanobium* sp. LEGE 10375 (5.49 × 10^8^ ± 1.34 × 10^8^ cells·cm^−2^; Figure 2(3a)). For *S. salina* LEGE 00041, the lowest number of biofilm cells was registered for the perspex (3.61 × 10^7^ ± 3.13 × 10^6^ cells·cm^−2^) and silicone hydrogel (4.11 × 10^7^ ± 3.81 × 10^6^ cells·cm^−2^) surfaces (Figure 2(1a)). These results suggested that silicone hydrogel is among the surfaces with fewer adhered cells.

Considering the biofilm wet weight (Figure 2b), *S. salina* LEGE 00041 showed no significant differences across the tested surfaces, with biofilms weighing about 30 mg on average (Figure 2(1b)). For *Cyanobium* sp. LEGE 06098, biofilms formed on epoxy-glass surfaces showed, on average, lower wet weight (28.6 ± 5.5 mg; Figure 2(2b)). In turn, *Cyanobium* sp. LEGE 10375 biofilms displayed, on average, lower wet weight when formed on glass (52.9 ± 4.3 mg; Figure 2(3b)).

Regarding the chlorophyll *a* production (Figure 2c), *S. salina* LEGE 00041 biofilms showed, on average, lower chlorophyll *a* content when formed on the silicone hydrogel (0.07 ± 0.04 µg·cm^−2^) than the rest of surfaces (Figure 2(1c)). Likewise, *Cyanobium* sp. LEGE 06098 and *Cyanobium* sp. LEGE 10375 biofilms (Figure 2(2c,3c)) produced, on average, a lower chlorophyll *a* amount on silicone hydrogel surfaces (0.48 ± 0.10 and 0.56 ± 0.25 µg·cm^−2^, respectively). These results are consistent with the number of biofilm cells.

Lastly, the thickness of *S. salina* LEGE 00041 biofilms (Figure 2(1d)) was equal for the tested surfaces. *Cyanobium* sp. LEGE 06098 biofilms showed lower thickness when formed on silicone hydrogel surfaces (41.3 ± 7.9 µm; Figure 2(2d)), while *Cyanobium* sp. LEGE 10375 biofilms were thinner on glass (74.1 ± 10.6 µm) and silicone hydrogel surfaces (84.8 ± 7.7 µm) (Figure 2(3d)).

### 3.3. Structure Analysis of Biofilms Developed on Tested Surfaces

**Figure 3:**
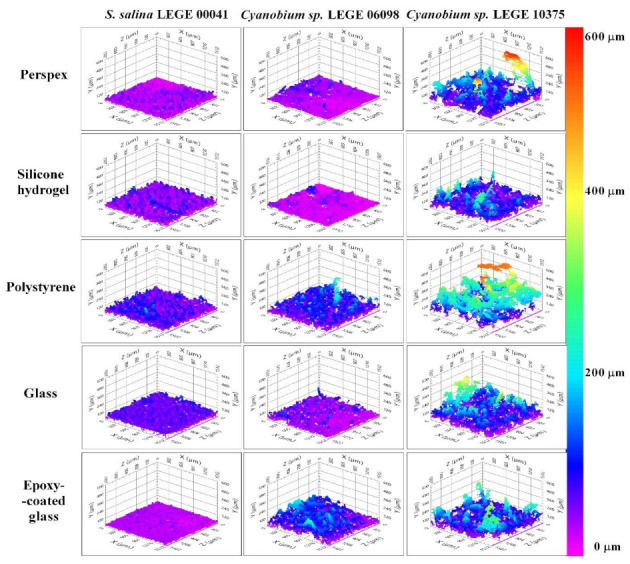
Representative 3D OCT images obtained for *S. salina* LEGE 00041, *Cyanobium* sp. LEGE 06098, and *Cyanobium* sp. LEGE 10375 biofilms formed on perspex, silicone hydrogel, polystyrene, glass, and epoxy-coated glass surfaces after 49 days. The color scale shows the range of biofilm thickness.

The study of biofilm structure deserves special attention since it indicates how cells interact with surfaces. Figure 3 shows representative 3D cross-sectional images obtained by OCT for cyanobacterial biofilms developed on the five surface materials. Cyanobacterial biofilms presented visible differences in their structure, while *S. salina* LEGE 00041 biofilms were more homogenous, *Cyanobium* sp. LEGE 06098 and *Cyanobium* sp. LEGE 10375 biofilms presented more heterogeneous contours, suggesting that cell–surface interactions depend on cyanobacterial isolates. Moreover, at the biofilm bottom of *S. salina* LEGE 00041, a uniform cell layer was observed, while *Cyanobium* sp. LEGE 10375 biofilms showed different shapes, and the uniform cell layer at the biofilm bottom was not detected.

Concerning the tested materials, biofilms formed on glass, epoxy-coated glass, or polystyrene surfaces presented a more developed structure for all cyanobacteria isolates than those developed on silicone hydrogel surfaces. This result is supported by the thickness of *Cyanobium* sp. LEGE 06098 and *Cyanobium* sp. LEGE 10375 biofilms.

**Figure 4:**
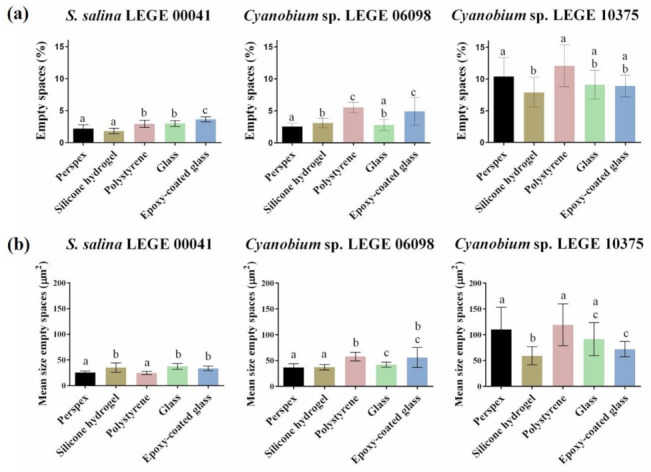
Mean percentage (**a**) and size (**b**) of empty spaces obtained for *S. salina* LEGE 00041, *Cyanobium* sp. LEGE 06098, and *Cyanobium* sp. LEGE 10375 biofilms developed on perspex ■, silicone hydrogel coating ■, polystyrene ■, glass ■, and epoxy-coated glass ■ surfaces after 49 days. For each cyanobacterial isolate, different lowercase letters indicate significant differences between surfaces with a confidence level greater than 95% (*p* < 0.05).

As the spatial confinement of microorganisms can influence biofilm formation [10], the percentage and the size of biofilm empty spaces were also determined. Figure 4a shows the mean percentage of empty spaces obtained for the different cyanobacterial biofilms formed on each surface. The mean percentage of empty spaces ranged from 1.8% (obtained from *S. salina* LEGE 00041 biofilm formed on silicone hydrogel) to 12.1% (obtained from *Cyanobium* sp. LEGE 10375 biofilm formed on polystyrene). The lowest values of empty spaces were observed for *S. salina* LEGE 00041 biofilms, whereas, in *Cyanobium* sp. LEGE 10375 biofilms, a higher percentage of empty spaces was detected. Additionally, a similar percentage of empty spaces was observed for *S. salina* LEGE 00041 biofilms formed on the different surfaces, from 1.8% to 3.7%. For *Cyanobium* sp. LEGE 06098 biofilms these values changed from 2.6% to 5.5%, and, for *Cyanobium* sp. LEGE 10375 biofilms, they changed from 7.9% to 12.1%. For *S. salina* LEGE 00041, biofilms formed on perspex and silicone hydrogel surfaces showed a lower percentage of empty spaces compared to other materials. *Cyanobium* sp. LEGE 06098 biofilms developed on perspex, silicone hydrogel, and glass surfaces revealed a lower percentage of empty spaces than on polystyrene and epoxy-coated glass surfaces, while *Cyanobium* sp. LEGE 10375 biofilms formed on the silicone hydrogel presented a lower percentage of empty spaces compared to perspex and polystyrene surfaces.

**Figure 5:**
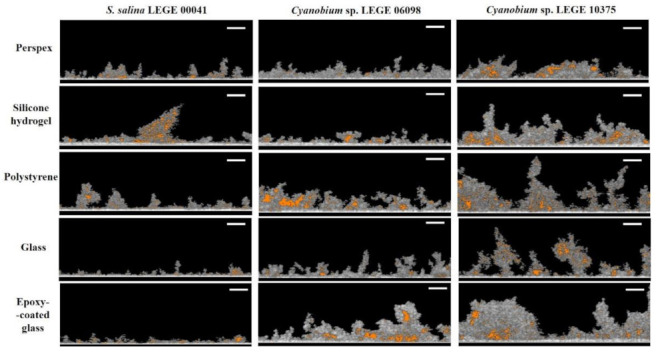
Representative 2D cross-sectional OCT images obtained for *S. salina* LEGE 00041, *Cyanobium* sp. LEGE 06098, and *Cyanobium* sp. LEGE 10375 biofilms formed on perspex, silicone hydrogel coating, polystyrene, glass, and epoxy-coated glass surfaces after 49 days. The empty spaces are indicated in orange (scale bars = 100 µm).

Figure 4b shows the mean size of empty spaces obtained for the different cyanobacterial biofilms. In addition, a graphical representation of the biofilm empty spaces for each surface and cyanobacteria strain is presented in Figure 5. The mean size of empty spaces ranged from 24 µm^2^ (obtained from *S. salina* LEGE 00041 biofilm formed on polystyrene) to 119 µm^2^ (obtained from *Cyanobium* sp. LEGE 10375 biofilm also formed on polystyrene). Regardless of the surface, the lower values of mean size of empty spaces were observed for *S. salina* LEGE 00041, and the higher values were observed for *Cyanobium* sp. LEGE 10375. In addition, *S. salina* LEGE 00041 biofilms displayed similar values of mean size of empty spaces (around 30 µm^2^) for glass, epoxy-coated glass, and silicone hydrogel surfaces (Figure 4b). In turn, *Cyanobium* sp. LEGE 06098 and *Cyanobium* sp. 10375 biofilms developed on silicone hydrogel surfaces showed, on average, a smaller size of empty spaces compared to glass, epoxy-coated glass, and polystyrene surfaces.

These results suggested that biofilm structure is not only dependent on the surface but also on the cyanobacterial isolate, as previously shown by Zheng et al. [33].

## 4. Discussion

In this study, the long-term performance of five different material surface characteristics in inhibiting biofilm formation by coccoid cyanobacteria was evaluated under hydrodynamic conditions found in marine environments, through an analysis of biofilm cell number, biofilm wet weight, chlorophyll *a* content, and biofilm thickness and architecture.

Since marine biofilm formation is influenced by several factors, including the surface properties and microfouler type [17,39], an extensive characterization of the surface materials and microorganisms was also performed in order to obtain more insight into the tested surfaces and their interactions with cyanobacterial isolates.

It is known that surface properties, including hydrophobicity and roughness, influence cell adhesion and subsequent biofilm formation [34,37]. In this study, the results from thermodynamic analysis classified glass as the most hydrophilic material, followed by epoxy-coated glass, perspex, and polystyrene surfaces (Table 1). Conversely, the silicone hydrogel surface was characterized as hydrophobic. These results are in accordance with previous studies [14,17,40]. In addition, the calculation of the free energy of adhesion indicates that cyanobacterial cell adhesion to glass and epoxy-coated glass is thermodynamically less favorable than to the silicone hydrogel coating, perspex, and polystyrene surfaces (Table 3). Concerning AFM analysis, results revealed that glass and perspex are the smoothest tested materials, displaying a lower *R_a_* value, followed by polystyrene and epoxy-coated glass surfaces (Table 1). In opposition, the silicone hydrogel surface showed higher roughness, as found in a previous study [17]. According to Dantas et al. [35], a reduction in surface roughness is directly related to a decrease in bacterial adhesion. Thus, both hydrophobicity and roughness results suggest that glass and epoxy-coated glass surfaces may be more efficient materials in controlling cyanobacteria biofilm formation than silicone hydrogel surfaces. Nevertheless, in general, the analysis of biofilm parameters indicated that cyanobacterial biofilms formed on glass and epoxy-coated glass surfaces were more developed than those formed on the silicone hydrogel coated surfaces (Figure 2(1–2a), (c), and (2d)). In fact, this adds to the debate on whether surfaces displaying higher degrees of hydrophobicity and roughness favor bacterial adhesion [41,42,43]. It has been shown, in particular for cyanobacterial adhesion, that it is not always possible to correlate surface hydrophobicity and roughness with cell attachment [17,44,45,46]. Furthermore, there is evidence that biofilm formation induces changes in the substratum surfaces since already attached cells modify surface properties [42,43,46,47].

The discrepancy observed between the material characterization and biofilm analysis may be explained by the formation of conditioning films resulting from the adsorption of molecules on the substrates that change the adhesion conditions for microorganisms [48,49]. The nature of formed films depends on the material type, surrounding environment, and microorganisms [44,48]. It is known that these conditioning films play an important role in cyanobacterial adhesion and subsequent biofilm formation [44]. Therefore, despite surface properties being of extreme importance, particularly during the adhesion phase [37], our results suggest that biofilm formation may also be modulated by other factors.

According to Zhang et al. [37], biofilm formation is also influenced by the physicochemical properties of the microorganisms. Indeed, the thermodynamic analysis of cyanobacterial cells indicated that *S. salina* LEGE 00041 is relatively more hydrophilic than *Cyanobium* sp. LEGE 06098 and *Cyanobium* sp. LEGE 10375 (Table 2). Moreover, the free energy of adhesion revealed that there is a tendency for *S. salina* LEGE 00041 to adhere less to all tested surfaces (Table 3). These results are corroborated by the biofilm parameter analysis, i.e., number of cells, chlorophyll *a* content, and biofilm thickness, which demonstrated that *S. salina* LEGE 00041 had a lower biofilm-forming capacity on these surfaces than the other cyanobacteria (Figure 2).

Concerning the performance of surface materials, the biofilm analysis indicated that cyanobacterial biofilms formed on glass and epoxy glass surfaces showed, on average, a higher number of *S. salina* LEGE 00041 and *Cyanobium* sp. LEGE 06098 cells compared to perspex, silicone hydrogel, and polystyrene surfaces. *Cyanobium* sp. LEGE 10375 biofilms formed on epoxy-coated glass exhibited a higher number of cells than the other surfaces, whereas the performance of perspex and polystyrene was different between cyanobacterial isolates. Furthermore, silicone hydrogel surfaces were among the surfaces that exhibited a low number of adhered cells (Figure 2a). Although these results were dependent on cyanobacterial isolates, they are supported by the literature. Glass, perspex, and polystyrene are not considered antifouling surfaces and are frequently used as positive fouling controls in several studies [50,51,52]. Likewise, epoxy resin coatings have not completely emerged in marine applications, especially where high fouling resistance is needed [53]. Lastly, silicone hydrogel surfaces are among the most successful antifouling coatings to prevent marine biofouling [54]. The commercial silicone hydrogel coating exerts a dual-mode of action, a ‘nonstick’ ability and a fouling-release effect, associated with relative higher elasticity. These features may decrease fouling settlements and cell cohesion interactions [17,55]. Additionally, this is a third-generation hydrogel-based fouling-release coating, which acquires a more hydrophilic behavior upon contact with water [56,57]. Thus, it can prevent either hydrophobic or hydrophilic interactions, delaying the adsorption of proteins, bacteria, and subsequent fouling [58,59,60,61].

Our results indicated that *S. salina* LEGE 00041 biofilms presented, on average, a similar wet weight for all tested surfaces, while *Cyanobium* sp. LEGE 06098 and *Cyanobium* sp. LEGE 10375 biofilms, formed on epoxy-coated glass and glass surfaces, respectively, exhibited a lower weight (Figure 2b). Indeed, once adhered, cells express different quorum sensing-related signaling molecules that stimulate or block EPS (extracellular polymeric substances) formation [62], which may justify the observed differences in wet weight concerning other biofilm parameters.

Regarding the chlorophyll *a* content, results were consistent with the number of biofilm cells (Figure 2a,c), which would be expected since several authors have proposed pigment quantification as a good indicator of cyanobacterial biofilm growth [63,64]. On the other hand, *S. salina* LEGE 00041 biofilms presented, on average, similar thickness values for all tested surfaces, while *Cyanobium* sp. LEGE 06098 biofilms formed on silicone hydrogel surfaces were thinner (supporting the biofilm cell number and chlorophyll *a* content) (Figure 2d). *Cyanobium* sp. LEGE 10375 biofilms developed on glass and silicone hydrogel surfaces showed a lower thickness than on the remaining surfaces (Figure 2d). Results demonstrated that there was no direct association between the number of biofilm cells and biofilm thickness. In fact, biofilm thickness, analogous to biofilm wet weight, is linked to several features of biofilm architecture, such as density, shape, and porosity, and cannot easily be isolated from environmental factors (e.g., flow, nutrient conditions, development age of the biofilm, carbon–nitrogen ratios, and temperature) [65].

Considering that heterogeneous structures may influence the biofilm resistance to mechanical and chemical challenges, such as fluid shear, detergents, and antifouling compounds [11], the study of biofilm architecture deserves special attention. Microorganisms often live in heterogeneous microenvironments with conditions that modulate local nutrient transport and chemical gradients, creating specialized niches for them. The spatial confinement of microorganisms can influence emergent phenomena, including quorum sensing, intracellular communication, and biofilm formation [10,66,67]. In fact, these microenvironment factors shape the structure of microbial communities and contribute to their phenotype diversity and synergism [10]. Our OCT analysis demonstrated that cyanobacterial biofilms presented visible differences in their structure; while *S. salina* LEGE 00041 biofilms were more homogenous, *Cyanobium* sp. LEGE 06098 and *Cyanobium* sp. LEGE 10375 biofilms presented more heterogeneous contours (Figure 3). Indeed, biofilms of *Cyanobium* sp. LEGE 10375 showed a higher biofilm wet weight and thickness when compared with *S. salina* LEGE 00041 (Figure 2b,d). Moreover, biofilms formed on the glass, epoxy-coated glass and polystyrene surfaces presented more developed structures, contrary to silicone hydrogel surfaces (Figure 3). These results are corroborated by the biofilm cell density.

In addition, the analysis of biofilm empty spaces demonstrated that *S. salina* LEGE 00041 biofilms showed lower percentage and mean size values of empty spaces compared to *Cyanobium* sp. LEGE 10375 (Figure 4). *S. salina* LEGE 00041 and *Cyanobium* sp. LEGE 06098 biofilms formed on silicone hydrogel surfaces showed, on average, a lower percentage of empty spaces compared to polystyrene and epoxy-glass surfaces (Figure 4a). Furthermore, *Cyanobium* sp. LEGE 10375 biofilms formed on silicone hydrogel surfaces showed, on average, a lower percentage of empty spaces compared to polystyrene (Figure 4a). While there were no significant differences in the size of empty spaces of *S. salina* LEGE 00041 biofilms formed on the glass, epoxy-coated glass, and silicone hydrogel surfaces, *Cyanobium* sp. LEGE 06098 and *Cyanobium* sp. LEGE 10375 biofilms developed on silicone hydrogel surfaces showed, on average, a lower size of empty spaces compared to polystyrene, glass, and epoxy-coated glass surfaces (Figure 4b). Overall, results from OCT analysis suggest that the biofilms formed on silicone hydrogel surfaces show, in general, a less developed and heterogeneous structure compared to polystyrene, glass, and epoxy-coated glass surfaces, while also presenting a low percentage and size of empty spaces. However, these results should be interpreted with caution as they vary among cyanobacterial isolates. According to Aufrecht et al. [10], the spatial distribution of microorganisms in their heterogenous network is determined by the EPS production ability and biofilm expansion over the biofilm formation process. In fact, biofilms developed on silicone hydrogel surfaces exhibited lower biofilm thickness, which may be related to their lower cellular growth and expansion.

## 5. Conclusions

Our results demonstrated high antifouling performance of the silicone hydrogel coating in inhibiting or delaying cyanobacterial biofilm formation. Additionally, the comprehensive analysis carried out in this study revealed that the surface material properties, together with the features of the fouling microorganisms, play a considerable role in marine biofouling.

## Figures and Tables

**Table 1:** The contact angles with water (*θ_w_*), formamide (*θ_F_*), and α-bromonaphthalene (*θ_B_*), hydrophobicity (according to Equation (4)) (∆*G*), and roughness (*R_a_*) determined for the tested surfaces. Values are presented as the mean ± standard deviation.

Surface	Contact Angle (°)			∆*G* (mJ·m^−2^)	*R_a_* (nm)
	*θ_w_*	*θ_F_*	*θ_B_*		
Perspex	72.6 ± 3.2	52.2 ± 3.2	22.4 ± 1.7	−42.7	6.2 ± 1.7
Silicone hydrogel	108.4 ± 3.5	104.0 ± 1.9	70.0 ± 2.0	−55.8	49.7 ± 8.3
Polystyrene	77.9 ± 3.6	62.1 ± 2.3	28.4 ± 2.6	−43.8	10.1 ± 2.2
Glass	27.8 ± 4.0	36.5 ± 3.9	44.3 ± 4.0	32.5	6.2 ± 0.9
Epoxy-coated glass	69.4 ± 3.0	56.8 ± 3.0	23.3 ± 2.2	−26.7	13.4 ± 4.1

**Table 2:** The contact angles with water (*θ_w_*), formamide (*θ_F_*), and *ɑ*-bromonaphthalene (*θ_B_*) and the hydrophobicity (∆*G*) for cyanobacterial strains, calculated according to Equation (4).

Microorganism	Contact Angle (°)			∆*G* (mJ·m^−2^)
	*θ_w_*	*θ_F_*	*θ_B_*	
*S. salina* LEGE 00041	32.3 ± 4.5	43.2 ± 5.1	45.5 ± 5.5	42.6
*Cyanobium* sp. LEGE 06098	23.4 ± 3.1	39.8 ± 5.3	36.0 ± 5.4	53.1
*Cyanobium* sp. LEGE 10375	41.7 ± 3.9	63.9 ± 3.9	33.3 ± 4.0	63.2

**Table 3:** Free energy of the interaction between cyanobacterial strains and tested surfaces (according to Equation (5)).

Microorganism	Δ*G^Adh^* (mJ·m^−2^)				
	Perspex	Silicone Hydrogel	Polystyrene	Glass	Epoxy-Coated Glass
*S. salina* LEGE 00041	2.4	0.5	4.5	38.5	12.3
*Cyanobium* sp. LEGE 06098	4.8	4.3	7.4	42.2	14.4
*Cyanobium* sp. LEGE 10375	6.8	6.4	9.7	46.6	18.3

Δ*G^Adh^*—free energy of adhesion.

## Data Availability

The data presented in this study are available on request from the corresponding author. The data will be publicly available when a public repository is selected.
